# Scrapping the Trained Nurse

**Published:** 1920-03-27

**Authors:** Gladys M. E. Leigh


### THE EDITOR'S LETTER-BOX.

(Correspondence on all subjects is invited, but we cannot in any way be responsible for the opinions expressed by our correspondents, who must give their name and address as a guarantee of good faith, but not necessarily for publication. Correspondents are reminded that brevity of style and conciseness of statement greatly facilitate early insertion.)

### Scrapping the Trained Nurse.

*To the Editor of* The Hospital.

Sir,—I have been profoundly interested in the articles entitled as above, which have appeared in the last two issues of The Hospital, especially as I now have lying in front of me Ierne's former articles "The Ministry of Health and the Nurse."

During November "Ierne" proudly admitted he held a brief for the Ministry of Health, now apparently the aforesaid brief has slipped from his fingers, and he stands desolate and alone. Formerly "Ierne" told us the Ministry of Health was willing and able to welcome the trained nurse within the fold, he also assured us that as the result of careful study he was personally convinced "that a hospital training was insufficient for the turning out of a successful health visitor," now he admits "that the privilege granted to a trained nurse of studying for one instead of two years at a polytechnic institute is of so little value that it is negligible," moreover he finds "an iron gate, locked and barred, with the trained nurse on the wrong side."

The salaries offered by the Ministry of Health are not sufficiently large to compensate trained nurses for the further financial outlay which they would incur as the result of the extra year's study, also from an economic standpoint, why spend five years working for qualifications which can be obtained as the result of two years' study?

Finally, no trained woman who upholds the dignity of her calling will squander the intrinsic value of a general training in work at which her value is rated on a par with first-year students who are taking a special course for two years only.—Yours faithfully,

Gladys M. E. Leigh.

